# The Impact of a New Anesthesiology Residency Program on the Number of Medical Students Matching Into Anesthesiology at a Single Institution: A Retrospective Longitudinal Study

**DOI:** 10.7759/cureus.50677

**Published:** 2023-12-17

**Authors:** Danielle Sawka, Abhishek Yadav, Mark Kendall, Matthew Diorio, Shyamal R Asher

**Affiliations:** 1 Anesthesiology, Brown University, Providence, USA; 2 Anesthesiology, Rhode Island Hospital, Brown University, Providence, USA

**Keywords:** residency program, anesthesia residency, residents in training, medical education, nrmp match, academic anesthesiology, anesthesiology

## Abstract

Introduction

There are projected workforce shortages within anesthesiology exacerbated by an increase in demand for anesthesia services and an aging anesthesia workforce. Given this mismatch, it is critical for the specialty to recruit the next generation of anesthesiologists and understand the factors affecting medical students’ decision to apply to anesthesiology. This study aims to evaluate the impact of establishing a new anesthesiology residency program at a single institution on the number of medical students that match into anesthesiology in the subsequent years.

Methods

A single-center, retrospective longitudinal study examined the number of medical students matching into anesthesiology at a single institution between 2013 and 2023, five years before and after the establishment of an accredited anesthesiology residency program. The data were compared to aggregated data on all US medical student applicants through the National Resident Matching Program.

Results

The pre-anesthesiology residency match rate (2013-2018) of medical students from Alpert Medical School (AMS) was 2.47% while the post-anesthesiology residency match rate (2019-2023) was 4.30%. This represents a 74% increase in the average proportion of medical students matching into anesthesiology after the start of the residency program compared to a 20% increase nationally over the same time period. The rate of change of AMS matched applicants after the implementation of the AMS anesthesia residency program increased compared to the national applicant pool (p= 0.002).

Conclusion

The establishment of a new accredited anesthesiology residency program increased the proportion of medical students matching into anesthesiology at the affiliated medical school in the subsequent five years. Exposure to an academic anesthesiology program improves medical student interest and ultimately matches rates in anesthesiology, a vital tool to address the projected shortages in the anesthesiology workforce.

## Introduction

According to the Association of American Medical Colleges (AAMC), by 2034, there is projected to be a shortage of 38,000 to 124,000 physicians [1]. Anesthesiologists are one important group of specialists included in this list. Due to the recent COVID pandemic, there was a surge in retirement among anesthesiologists, along with an expansion of anesthesia roles in the non-operative setting. This trend has exacerbated the current workforce shortage and will likely continue to increase the need for more anesthesia providers in the future [2]. Given the increasing demand for anesthesiologists, it is critical to understand the driving factors that shape medical students’ decision to apply to anesthesiology.

Exposure to the field of anesthesiology and connecting with peer mentors are vital factors in gaining medical student interest. Medical students have limited exposure to anesthesiology prior to their clerkship rotations [3,4]. Among a sample of accredited US anesthesiology residency programs, only 43% reported anesthesiology faculty teaching medical students during their preclinical years [5]. In a 2020 survey, about 80% of responding medical schools did not require medical students to take a clinical rotation in anesthesia [6]. The availability of mentors is also formative for medical students in choosing their career path in academic medicine. Across the globe, the presence of a mentor has been a significant independent factor among medical students in choosing a residency program in anesthesiology [7,8]. For example, in Rwanda, a country with a dire shortage of anesthesiologists, ineffective mentorship was cited as one of the top reasons for discouraging medical students from pursuing this specialty [9]. However, despite mentorship becoming a core component of residency training programs, its prevalence in anesthesia is unknown. 

There remains a dearth of literature analyzing the impact of residents on medical student career choices. To our knowledge, studies investigating the impact of a new physician residency program have mainly focused on its effects on patient care and hospitals, not future physicians [10-12]. The absence of an anesthesiology residency program at a medical school may be detrimental to medical education. Medical students may have reduced exposure to the clinical teaching experience, lack of faculty and resident mentorship, and understanding of the field of anesthesiology. This may adversely affect a medical student’s decision to ultimately pursue a career in anesthesiology.

The Warren Alpert Medical School of Brown University (AMS) is the only medical school in the US state of Rhode Island. Before 2018, there was no Accreditation Council for Graduate Medical Education (ACGME)-accredited anesthesiology residency program in Rhode Island. In July 2018, the first class of the ACGME-accredited anesthesiology program enrolled at AMS and their affiliated Lifespan hospitals. There was a steady increase in the total number of anesthesiology residents from 13 in 2018 to a total of 56 residents across three classes in 2023. AMS medical students who rotated through anesthesiology electives had limited direct exposure to anesthesiology residents and its educational curriculum prior to 2018. However, AMS medical students who rotated through anesthesiology electives in subsequent years were exposed to anesthesiology residents and the residency educational curriculum including daily intraoperative teaching and a formal didactic curriculum.

Anesthesiologists in the United States enter the subspecialty by participating in the National Resident Matching Program (NRMP). The aim of this study is to investigate the impact of establishing the first accredited anesthesiology residency program at AMS on the number of AMS matriculating medical students who successfully match into an anesthesiology residency program in the subsequent years. The total number of US medical students who matched into anesthesiology residency programs was also investigated over the same time period.

## Materials and methods

This single-center, retrospective longitudinal study was performed using medical students from AMS and compared to aggregated data on all US medical student applicants publicly provided from the NRMP website. The Rhode Island Hospital Institutional Review Board determined that this study is exempt from human subject research under 45 Code of Federal Regulations 46.104(d) requirements (IRB# 1976869).

The number of AMS medical students who successfully matched into an anesthesiology residency program from 2013 to 2023 was extracted from the AMS Residency Match results [13]. In addition, the total number of matriculating AMS medical students who successfully matched into residency programs of all medical specialties was also extracted from the AMS Residency Match website.

The NRMP is a public data resource that annually reports the number of positions offered and successfully filled for all residency programs participating in the residency match process [14]. The number of applicants who successfully matched into an anesthesiology residency program from 2013 to 2023 was extracted from the NRMP database [15]. The total number of medical students who successfully matched into residency programs of all medical specialties in the United States was extracted from the same NRMP data source.

Descriptive statistics were used to describe the number of AMS matched applicants as well as the number of anesthesiology residency programs that filled in the NRMP data. We performed linear regression analysis to identify whether an anesthesia residency program increased the rate of match rate percentages compared to the rate of increase pre-anesthesia residency program. All analyses were performed using SAS software version 9.4 (SAS Institute Inc., Cary, North Carolina).

## Results

A total of 11 years of NRMP and AMS match data from 2013 to 2023 was included in the analysis. A total of 44 AMS medical students and 9,859 applicants were identified as having matched into the anesthesiology program during the study period. The AMS match rate into anesthesiology consistently increased from 2013 when there were no applicants to a peak of 6.25% in 2022 (Table 1). When compared to the NRMP data, the match rate of all applicants in the United States was 4.56% peaking at 6.50% in 2023 (Table 2).

**Table 1:** Alpert Medical School graduating medical students who matched into an anesthesiology residency program and all medical specialties in the US from 2013 to 2023 Data obtained from the AMS Residency Match. AMS = Alpert Medical School.

Year	AMS medical students		Percent
	Matched into an anesthesiology residency	Matched into all medical residencies	
2013	0	105	0.00
2014	2	91	2.20
2015	4	102	3.92
2016	2	113	1.77
2017	4	113	3.54
2018	4	117	3.42
2019	3	125	2.40
2020	4	132	3.03
2021	5	113	4.42
2022	9	144	6.25
2023	7	127	5.51

**Table 2:** Applicants who matched into an anesthesiology residency program in the National Residency Matching Program from 2013 to 2023 Data obtained from the National Residency Matching Program [13].

Year	National applicants		Percent
	Matched into an anesthesiology residency	Matched into all medical residencies	
2013	748	16390	4.56
2014	754	16399	4.60
2015	799	16932	4.72
2016	774	17057	4.54
2017	803	17480	4.59
2018	861	17740	4.85
2019	907	17763	5.11
2020	936	18108	5.17
2021	1024	18435	5.55
2022	1054	18486	5.70
2023	1199	18498	6.48

**Figure 1:**
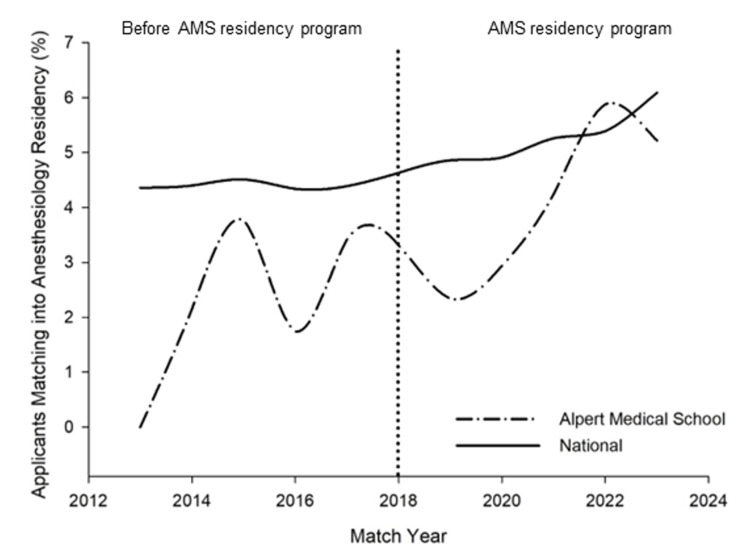
Alpert Medical School students that matched into anesthesiology compared to the total national applicants. Dotted vertical line = Initiation of the Alpert medical school anesthesiology residency program. AMS = Alpert Medical School.

Figure 1 illustrates the number of medical students that matched into anesthesiology as a percentage of the total number of applicants in a particular year for both AMS and NRMP groups (Pearson's R = 0.75, p = 0.008). The match rate prior to the initiation of the AMS anesthesiology residency program (2013-2018) was 2.47% whereas the total number of applicants applying to anesthesiology residency during the same period was 4.64%. After the commencement of the AMS anesthesiology program (2019-2023), the match rate of applicants at AMS was 4.30% and nationally was 5.60% during the same time period.

After the start of the residency program, the AMS match rate in an anesthesiology residency program has been increasing at a faster rate than the national average (Table 3). At AMS, there was a 74% increase in the average proportion of students that matched into anesthesiology residency in 2019-2023 compared to that before the start of the AMS residency program. During this same period, the national average proportion of students matching into anesthesiology residency increased only by 20%. In 2023, the proportion of AMS students matching in anesthesiology residency is now more in line with the national average five years after the establishment of an anesthesiology residency program.

**Table 3:** The rate of change of AMS matched applicants before and after the implementation of the AMS anesthesia residency program compared to the total applicants for the same time period. ^†^Statistically significant. AMS = Alpert Medical School.

Statistic	Alpert Medical School students		National applicants	
	2013-2018	2019-2023	2013-2018	2019-2023
Rate of change	-0.02	0.56	0.03	0.41
p-Value	0.913	0.002^†^	0.802	0.073
Difference in rate of change	-0.57		-0.39	
p-Value	0.036^†^		0.303	

## Discussion

This study aimed to evaluate how establishing a new anesthesiology residency program at AMS-affiliated hospitals has impacted match rates of medical students at AMS into anesthesiology. The state of Rhode Island is unique in that there is only one accredited medical school and only one accredited anesthesiology residency program. Therefore, by comparing the match rates in anesthesiology before and after the establishment of the new residency program, we attempted to identify the impact of the new residency program on AMS medical student career choices.

It was interesting to see an increase in the number of medical students matching into an anesthesiology residency program, particularly during the COVID-19 pandemic. In the past several years, there has been an increase in the number of anesthesia programs established in the United States. The increase in anesthesia programs may provide the necessary relief for the projected shortage of anesthesiologists. In addition, anesthesia programs have increased their presence on social media platforms allowing potential residents to evaluate residency programs virtually. Since the establishment of the anesthesiology residency program, the number of medical students in AMS matching into anesthesiology residency has significantly increased at a greater rate than the national average. Our findings suggest that the presence of an anesthesiology residency program at AMS has positively influenced the career decisions of AMS medical students.

Before 2018, AMS medical students who rotated through the anesthesiology elective received an equivalent clinical anesthesiology exposure but had minimal interactions with anesthesiology residents and the educational environment that goes along with a residency program. After 2018, AMS medical students who rotated through the anesthesiology elective had the opportunity to work one-on-one with anesthesiology residents and participate in intraoperative teaching and attend formal lectures associated with the anesthesia residency program. Anesthesiology residents engaged further with the medical school after the establishment of an Anesthesiology Interest Group and an Anesthesiology Preclinical Elective. These extracurricular activities provide AMS students with broader exposure to the field of anesthesiology including resident’s perspectives outside their clerkship experience.

Additionally, AMS medical students were likely more comfortable learning vital skills such as IV access, ultrasound assessment, and airway management from residents. This is not surprising since it has been estimated that one-third of medical students learning in the clinical setting comes from residents [16]. Resident teaching takes a different form that is complementary to the teaching from attending. Residents are near-peers and thus can connect with students in a different way and tend to teach medical students about the practical skills of patient management rather than focus on factual knowledge [17]. Resident teaching and mentorship have been shown to have a strong impact on medical students’ perception of the quality of a rotation [18] and their career choice [19]. Opportunities to interact with anesthesiology residents likely provided a better understanding of the quality of life, day-to-day practice and educational opportunities in anesthesiology residency, which are increasingly critical factors that medical students use when making a specialty selection. AMS students were able to obtain mentorship more easily for academic projects in anesthesiology and letters of recommendation from residents and attendings in an educational environment of a residency program. All of these factors likely contributed to a positive medical student experience and an increased interest in applying for an anesthesiology residency.

This study has limitations including the examination of a single medical school and medical center. In addition, the study is retrospective in nature and thus there may have been other factors influencing medical student specialty choice that are not accounted for by our analysis. For example, a change in market conditions for a specific specialty may be a reason that influences their decision. The COVID-19 pandemic may have also affected students’ decisions on the choice of medical specialty due to the inability to explore specialties of interest, improve their residency application, or obtain recommendation letters [20]. Future studies investigating factors that influence medical students' career choices during medical school may provide valuable information affecting decision-making and specialty preference and are warranted.

## Conclusions

The start of a new anesthesiology residency program increased the proportion of medical students matching into anesthesiology at the affiliated medical school in the subsequent five years. Our results support the idea that exposure to an academic anesthesiology program improves medical student interest and ultimately matches rates in anesthesiology. The presence of residents to serve as mentors within the anesthesiology specialty may be an important reason why medical students choose a particular career path. With the projected shortages in anesthesiology staff, it is important to provide medical students an early exposure to academic anesthesiology programs to increase their interest in this field.
